# Systematic Analysis of Non-coding RNAs Involved in the Angora Rabbit (*Oryctolagus cuniculus*) Hair Follicle Cycle by RNA Sequencing

**DOI:** 10.3389/fgene.2019.00407

**Published:** 2019-05-03

**Authors:** Bohao Zhao, Yang Chen, Shuaishuai Hu, Naisu Yang, Manman Wang, Ming Liu, Jiali Li, Yeyi Xiao, Xinsheng Wu

**Affiliations:** ^1^College of Animal Science and Technology, Yangzhou University, Yangzhou, China; ^2^Joint International Research Laboratory of Agriculture and Agri-Product Safety, Yangzhou University, Yangzhou, China

**Keywords:** rabbit, non-coding RNA, sequencing, hair follicle cycle, ceRNA

## Abstract

The hair follicle (HF) cycle is a complicated and dynamic process in mammals, associated with various signaling pathways and gene expression patterns. Non-coding RNAs (ncRNAs) are RNA molecules that are not translated into proteins but are involved in the regulation of various cellular and biological processes. This study explored the relationship between ncRNAs and the HF cycle by developing a synchronization model in Angora rabbits. Transcriptome analysis was performed to investigate ncRNAs and mRNAs associated with the various stages of the HF cycle. One hundred and eleven long non-coding RNAs (lncRNAs), 247 circular RNAs (circRNAs), 97 microRNAs (miRNAs), and 1,168 mRNAs were differentially expressed during the three HF growth stages. Quantitative real-time PCR was used to validate the ncRNA transcriptome analysis results. Gene ontology (GO) enrichment and Kyoto Encyclopedia of Genes and Genomes (KEGG) pathway analyses provided information on the possible roles of ncRNAs and mRNAs during the HF cycle. In addition, lncRNA–miRNA–mRNA and circRNA–miRNA–mRNA ceRNA networks were constructed to investigate the underlying relationships between ncRNAs and mRNAs. LNC_002919 and novel_circ_0026326 were found to act as ceRNAs and participated in the regulation of the HF cycle as miR-320-3p sponges. This research comprehensively identified candidate regulatory ncRNAs during the HF cycle by transcriptome analysis, highlighting the possible association between ncRNAs and the regulation of hair growth. This study provides a basis for systematic further research and new insights on the regulation of the HF cycle.

## Introduction

Hair follicle (HF) development is a complex morphogenetic process that relies on a variety of signaling systems, and on interactions between mesenchymal and epithelial tissues (Hardy, 1992; Oro and Scott, 1998). Under the biological regulation of stem cells, mature HFs undergo a cycling and continuous self-renewal process, with periods of active growth (anagen), followed by regression (catagen), and rest (telogen) (Cotsarelis et al., 1990; Paus and Cotsarelis, 1999; Fuchs and Segre, 2000; Oshima et al., 2001). In murine HF cycling, key parameters for the recognition of distinct stages have been defined in many studies (Chase et al., 1951; Chase, 1954; Straile et al., 2010). Moreover, the immediate removal of hair shafts could induce homogeneous anagen development in the murine model, which leads to the spontaneous entering of consecutive stages (catagen and telogen). In this way, the methods for the analysis of murine HF growth were provided, and were based on histologic and ultrastructural studies on murine hair cycling (Veen et al., 1999; Müller-Röver et al., 2001). During the anagen phase, the hair root is dividing and adding to the hair shaft. The HFs actively grow, surrounded by dermal fibroblasts that have not reached the subcutis. During the catagen phase, interfollicular dermal fibroblasts fully surrounded the HFs, the blood supply is cut off, and the hair bulb starts to atrophy. Finally, HFs enter the telogen phase, where hair shafts stop growing, and begin to fall due to synthesis and release of hair cycle inhibitor (Stenn and Paus, 2001). The molecular mechanisms underlying the regulation of the hair cycle and of HF development are of interest in medicine and developmental biology (Shirokova et al., 2016; Ahmed et al., 2017; Sardella et al., 2017).

Long non-coding RNAs (lncRNAs), microRNAs (miRNAs), and circular RNAs (circRNAs) are non-coding RNA (ncRNA) that are not translated into proteins but regulate many cell functions and play vital roles in many biological processes (Mattick and Makunin, 2006; Guttman and Rinn, 2012). miRNAs are small ncRNA molecules (∼22 nucleotides length) that repress gene expression by recognizing specific target mRNAs (Ding et al., 2009). An increasing number of studies reported that lncRNAs (non-coding RNAs containing more than 200 base pairs) regulate interactions between genes and proteins, act as decoys that bind to miRNAs or proteins, or bind to enhancer regions or neighboring loci to modulate the transcription of their target gene as enhancers (Winkle et al., 2015; Chen et al., 2016; Li et al., 2016; Song et al., 2017; Lu et al., 2018). CircRNAs consist of continuous loop structures, are more stable than linear mRNAs, and are conserved between different species (Stoffelen et al., 2012; Memczak et al., 2014). As sponges for miRNAs, circRNAs act as competitive inhibitors that interfere with the binding of miRNAs to their target genes (Hansen et al., 2013; Zhong Z. et al., 2016). circRNAs may also regulate the function of RNA-binding proteins and the transcription activity of the host gene (Reut et al., 2014; Li et al., 2015). Although circRNAs have been categorized as ncRNA, they have been reported to have the ability to code proteins as gene regulators (Pamudurti et al., 2017).

Accumulating evidence suggests that lncRNAs are involved in the regulation of the HF cycle (Wang et al., 2017; Song et al., 2018; Zhu Y.B. et al., 2018). Specific lncRNAs, such as HOTAIR, H19, and RP11-766N7.3, have been reported to be differentially expressed in dermal papilla cells after Wnt signaling by using lncRNA microarrays, and integrated analysis by RNA-seq techniques has led to the identification of potential lncRNA, which may play a role during the initiation of secondary HFs (Lin et al., 2015; Yue et al., 2016). Moreover, aberrantly expressed miRNAs may participate in the regulation of the development of skin and HFs. miRNAs play important roles in several signaling pathways and control gene expression patterns during the HF cycle (Mardaryev et al., 2010; Chao et al., 2013; Ahmed et al., 2014; Zhou et al., 2018). In addition, the expression levels and functions of circRNAs associated with skin color during different skin differentiation stages have been analyzed by RNA-seq (Zhu Z. et al., 2018).

However, only very few studies have systematically investigated ncRNAs during the HF cycle. This study established a HF cycle synchronization model in the rabbit, allowing an integrated analysis of ncRNAs and mRNAs expressed during the different HF cycle phases (anagen, catagen, and telogen). Numerous essential factors related to the HF cycle have been uncovered, contributing to the understanding of HF cycle regulation and suggesting new potential therapies for hair-related diseases.

## Materials and Methods

### Animals

Twelve 6-month-old male Wanxi Angora rabbits were used to establish the HF synchronization model. They were all housed under the same conditions, including temperature, and were fed the same diet (feed pellet and grass). Animals were reared in a controlled environment and had the same length of the hair coat phenotypes. The experimental procedures in this study were approved by the Animal Care and Use Committee of Yangzhou University.

To estimate the wool growth rate and to determine the onset of the anagen phase, the dorsal area of experimental animals was shaved with electronic clippers and entry into anagen was determined by the appearance of light pink skin and by hair regrowth. The length of the hair coat was measured, skin samples were collected after shaving, samples were fixed in 4% formaldehyde, and paraffin sections were stained with hematoxylin–eosin (HE) for histological observations. Longitudinal sections of the HFs showed the skin status and the phase of the HF cycle.

### Tissue Collection

Rabbits were anesthetized via ear vein injections of 0.7% pentobarbital sodium (6 mL/kg), dorsal skin samples (1 cm^2^) were collected, and placed immediately in liquid nitrogen for RNA extraction. Iodine solution was applied on the wound to prevent bacterial infection. Samples were harvested at different phases of the HF cycle for gene expression profiling: growth (anagen), cessation (catagen), and rest (telogen). Three sample replicates were collected at days 90, 130, and 150 of the HF cycle for ncRNA and mRNA sequencing analysis.

### RNA Isolation and RNA Quantification

Total RNA from nine samples was extracted from skin tissue using Trizol reagent (Invitrogen, Carlsbad, CA, United States), according to the manufacturer’s instructions. RNA degradation and contamination were monitored by running samples on 1% agarose gels. RNA purity was analyzed via a NanoPhotometer^®^ spectrophotometer (IMPLEN, CA, United States). RNA concentration was measured using the Qubit^®^ RNA Assay Kit and a Qubit^®^ 2.0 Fluorometer (Life Technologies, CA, United States). RNA integrity was assessed via the RNA Nano 6000 Assay Kit and a Bioanalyzer 2100 system (Agilent Technologies, CA, United States). lncRNAs and miRNAs were quantified following the same procedure used for conventional mRNAs. Quantification of circRNAs was performed adding an exonuclease to degrade non-circRNAs. Briefly, two samples containing the same amount of RNA were collected. In one sample, linear RNA was digested with RNase R (Cat. No. RNR07250, Epicentre Company, United States), leaving only the circRNAs, while the other sample was not treated with RNase R. The two RNA samples were reverse transcribed. The samples subjected to RNase treatment were used to detect circRNAs, whereas the untreated samples were used to detect β-actin.

### Library Construction for lncRNA and circRNA Sequencing

A total amount of 3 μg of RNA per sample was used for lncRNA sequencing and of 5 μg for circRNA sequencing. First, ribosomal RNAs were removed with the Epicentre Ribo-zero^TM^ rRNA Removal Kit (Epicentre, United States) and the rRNA-depleted samples were purified by ethanol precipitation. Subsequently, sequencing libraries were generated using the rRNA-depleted RNA and the NEBNext^®^ Ultra^TM^ Directional RNA Library Prep Kit for Illumina^®^ (NEB, United States), following the manufacturer’s recommendations. First strand cDNA was synthesized using random hexamer primers and M-MuLV Reverse Transcriptase (RNaseH). Second strand cDNA synthesis was performed using DNA Polymerase I and RNase H. After adenylation of the 3′ ends of DNA fragments, NEBNext Adaptor with hairpin loop structure were ligated to prepare for hybridization. To select cDNA fragments with a preferential length of 150∼200 bp, the library fragments were purified with the AMPure XP system (Beckman Coulter, Beverly, MA, United States). Then, 3 μl of USER Enzyme (NEB, United States) was used with size-selected, adaptor-ligated cDNA before the PCR. Finally, the PCR products were purified (AMPure XP system) and the library quality was assessed with the Agilent Bioanalyzer 2100 system.

### Library Construction for Small RNA Sequencing

A total amount of 3 μg of RNA per sample was used as input material for the small RNA library. Sequencing libraries were generated using the NEBNext^®^ Multiplex Small RNA Library Prep Set for Illumina^®^ (NEB, United States), following the manufacturer’s recommendations. Briefly, NEB 3′ SR Adaptor was directly and specifically ligated to the 3′ end of miRNAs, siRNAs, and piRNAs. After the 3′ ligation reaction, the SR RT Primer was hybridized to the excess of 3′ SR Adaptor, transforming the single-stranded DNA adaptor into a double-stranded DNA molecule. Then, the 5′ ends adapter was ligated to the 5′ ends of the miRNAs, siRNAs, and piRNAs. First strand cDNA was synthesized using M-MuLV Reverse Transcriptase (RNase H–). DNA fragments of 140–160 bp length (the length of small non-coding RNAs plus the 3′ and 5′ adaptors) were recovered and dissolved in 8 μL of elution buffer. Finally, the library quality was assessed using the Agilent Bioanalyzer 2100 system and DNA High Sensitivity Chips.

### Clustering and Sequencing of lncRNAs, circRNAs, and miRNAs

Clustering of the index-coded samples was performed on a cBot Cluster Generation System using TruSeq PE Cluster Kit v3-cBot-HS (Illumina), according to the manufacturer’s instructions. After cluster generation, the lncRNA and circRNA libraries were sequenced on an Illumina Hiseq 4000 platform and 150 bp paired end reads were generated. The miRNA library was sequenced on an Illumina Hiseq 2500 platform and 50 bp single-end reads were generated.

### Quality Control

For lncRNA and circRNA sequencing, raw data (raw reads) in fastq format were first processed through in-house perl scripts. In this step, clean data (clean reads) were obtained by removing reads containing adapter, reads containing ploy-N, and low-quality reads from the raw data. For miRNA sequencing, raw data (raw reads) in fastq format were first processed through custom perl and python scripts. In this step, clean data (clean reads) were obtained by removing reads containing ploy-N, with 5′ adapter contaminants, without 3′ adapter or the insert tag, containing ploy A or T or G or C, and low-quality reads from raw data. At the same time, the Q20, Q30 scores, and GC-content of the raw data were calculated. A specific length range from the clean reads was selected to conduct all the downstream analyses, based on clean data of high quality.

### Genome Mapping, Transcriptome Assembly, and ncRNAs Identification

For lncRNA and circRNA sequences, the reference genome (*Oryctolagus cuniculus* genome obtained from Ensembl OryCun2.0) and annotation files were directly downloaded from the genome website. An index of the reference genome was built using bowtie2 (Langmead and Salzberg, 2012), and paired-end clean reads were aligned to the reference genome using HISAT2 v2.0.4 (Pertea et al., 2016). Also, the small RNA tags were mapped to the reference sequence with bowtie2 (Langmead and Salzberg, 2012) without mismatch to analyze the expression and distribution of miRNA sequences in the reference genome.

The mapped lncRNA and mRNA reads from each sample were assembled by means of StringTie (v1.3.1) (Pertea et al., 2016), following a reference-based approach. The circRNAs were detected and identified using find_circ (Memczak et al., 2014). Alignment of the small RNA tags to miRBase20.0 identified known *Oryctolagus cuniculus* and *Mus musculus* (near-source species) miRNAs. Mirdeep2 software (Friedländer et al., 2011) was used to identify potentially novel miRNAs and to draw the secondary structures and the characteristics of the hairpin structures of miRNA precursors.

### Quantification of lncRNA, circRNA, mRNA, and miRNA Expression Levels

Cuffdiff (v2.1.1) was used to calculate fragments per kilo-base millions of exon per million fragments mapped (FPKM) of both lncRNAs and mRNA in each sample (Trapnell et al., 2010). FPKMs of genes were computed by summing the FPKMs of transcripts in each gene group. Also, the raw counts were first normalized using transcripts per million (TPM) (Zhou et al., 2010) and normalized expression levels = (read count^∗^1,000,000)/lib size (lib size is the sum of circRNA read counts). This was used to determine the circRNA expression levels. On the other hand, miRNA expression levels were estimated by TPM based on the following criteria: Normalization formula: Normalized expression = mapped read count/total reads^∗^1,000,000. The differential expression of ncRNAs was determined using the DESeq R package (1.10.1) (Wang et al., 2010).

### Target Gene Prediction, GO, and KEGG Enrichment Analysis

In *cis* regulation, lncRNAs can act on neighboring target genes. Coding genes 10 k/100 k upstream or downstream of the lncRNA gene were searched for and their function was analyzed. For *trans* regulation, lncRNAs and their target genes were analyzed based on their expression levels. The correlation between lncRNAs and coding gene expression levels were calculated with custom scripts; then, the genes from different samples were clustered using WGCNA (Langfelder and Horvath, 2008) to search for common expression modules and to analyze the function via functional enrichment analysis. The target genes of miRNAs and miRNA target sites in exons of circRNA loci were identified using miRanda (version 3.3a, main parameter: -sc 140; -en -10; -scale 4; -strict) (Enright et al., 2004). Differentially expressed (DE) ncRNAs were annotated by gene ontology (GO) enrichment and Kyoto Encyclopedia of Genes and Genomes (KEGG) pathway analyses to investigate their biological functions. Briefly, GO analysis was applied to elucidate genetic regulatory networks of interest by forming hierarchical categories according to the molecular function (MF), cellular component (CC), and biological process (BP) aspects of the differentially expressed genes^1^. KEGG pathway analysis was performed to explore the significantly enriched pathways of DE genes^2^.

### Quantitative Real-Time PCR

Eight mRNAs, four lncRNAs, and five circRNAs associated with skin and the HF cycle were selected for validation by qRT-PCR analysis. Approximately 1μg of total RNA was used to synthesize cDNA using HiScript II Q Select RT SuperMix for qPCR (Vazyme). qRT-PCR was performed using the AceQ qPCR SYBR^®^ Green Master Mix (Vazyme), according to the manufacturer’s instructions, and data were analyzed via QuantStudio^®^ 5 (Applied Biosystems). The specific primer sequences are listed in Supplementary Table S1. The expression levels were calculated using the 2^-ΔΔCt^ method (Schmittgen and Livak, 2008), with glyceraldehyde 3-phosphate dehydrogenase (GAPDH) as reference gene.

To confirm the miRNA transcriptome data, three miRNAs were selected for qRT-PCR analysis. Approximately 2μg of total RNA was used to synthesize cDNA after adding a poly (A) tail to the 3′ end of the miRNAs using the miRcute Plus miRNA First-Strand cDNA Synthesis Kit (Tiangen). qRT-PCR was performed using the miRcute miRNA qPCR Detection Kit (SYBR Green), according to the manufacturer’s instructions. The specific primers were designed by Beijing Tiangen Co., Ltd. and the product code sets are listed in Supplementary Table S1. The U6 small nuclear RNA gene was chosen as internal control. The expression levels were calculated using the 2^-ΔΔCt^ method (Schmittgen and Livak, 2008), and the results of the experiments were normalized to the expression levels of the constitutively expressed U6 gene.

### Construction of ncRNAs Regulatory Networks

To investigate the role and interactions between ncRNAs and mRNAs during the HF cycle, ncRNAs regulatory networks were constructed. For the interaction network of lncRNA–miRNA, DE lncRNAs were filtered out according to the homology between lncRNA and miRNA precursor; then, the targeted relationships between lncRNA and miRNA were predicted by miRanda. Then, the regulatory networks of lncRNA–miRNA–mRNA pairs and circRNA–miRNA–mRNA pairs were constructed according to the following steps: (i) the ncRNAs and mRNAs that were upregulated or downregulated were retained; (ii) the interactions of lncRNA–miRNA, miRNA–mRNA, and miRNA–circRNA were predicted by miRanda, which predicts miRNA binding seed sequence sites, as well as overlapping the same miRNA binding site in lncRNAs, circRNAs, and mRNAs; (iii) The lncRNA–miRNA–mRNA pairs network covered two cases: one was the upregulated lncRNA-downregulated miRNA-upregulated mRNA, the other was the downregulated lncRNA-upregulated miRNA-downregulated mRNA. The circRNA–miRNA–mRNA pairs network covered two cases: one was the upregulated circRNA-downregulated miRNA-upregulated mRNA, the other was the downregulated circRNA-upregulated miRNA-downregulated mRNA. Cytoscape software was used to build and visually display the networks.

### Luciferase Assay

The dual-luciferase reporter system E1910 (Promega, Madison, WI, United States) was used to perform luciferase activity assays. The miR-320-3p mimic and miR-320-3p negative control mimics were purchased from Shanghai GenePharma Co., Ltd. Wild-type luciferase reporter vectors (pMir-HTATIP2-3’UTR-WT, pMir-LNC_002919-WT, and pMir-novel_circ_0026326-WT) were constructed using the primers shown in Supplementary Table S2. Their substitution mutants (pMir-HTATIP2-3’-UTR-MUT, pMir-LNC_002919-MUT, and pMir-novel_circ_0026326-MUT) were synthesized by Beijing Tsingke Co., Ltd. Briefly, the skin fibroblast cells of rabbit (RAB-9, ATCC^®^ CRL-1414^TM^) were cultured in 24-well tissue culture plates. Cells were co-transfected with the pMir-report luciferase reporter, the miRNA (miR-320-3p) mimics and pRL-TK using Lipofectamine^TM^ 2000 (Invitrogen). After 48 h of culture at 37°C, transfected cells were lysed with 100 μl of passive lysis buffer. Next, 20 μl of lysates were mixed with 100 μl of LAR II, and firefly luciferase activity was measured by using a luminometer. As an internal control, 100 μl of Stop & Glo reagent was added to the sample. Firefly luciferase activity was normalized to the corresponding Renilla luciferase activity.

## Results

### Hair Follicle Cycle Synchronization Model

**FIGURE 1:**
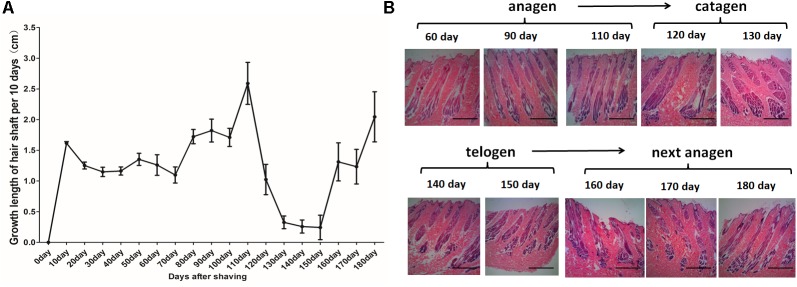
Hair follicle synchronization model in Angora rabbits. **(A)** Measurement of length of the hair coat after shaving the dorsal area of Angora rabbits. **(B)** HE staining of sequential skin samples after shaving the dorsal area of rabbits. The anagen, catagen, telogen, and subsequent anagen stages were determined based on the histomorphology of hair follicles.

For the HF cycle synchronization model, Angora rabbits were used. The obtained observations showed that the length of the hair coat increased steadily until day 110. Between days 120 and 150, the growth rate of wool declined rapidly. Then, between days 160 and 180, the wool recovered and once again showed an increased growth rate (Figure 1A). Histological analysis showed rapid growth of the hair shaft and increasing depth of the HF between days 0 and 110. Then, the growth of the hair shaft and the depth of the HF decreased between days 120 and 130. Finally, the hair shaft started to fall off and the hair bulbs atrophied between days 140 and 150. After the HF cycle ended, a new HF appeared, the growth of the hair shaft recovered and the HFs moved into a new cycle (Figure 1B). In conclusion, the hair cycle of Angora rabbits is characterized by an anagen phase between days 0 and 110, a catagen phase between days 120 and 130, and a telogen phase between days 140 and 150.

### Differentially Expressed lncRNAs, mRNAs, miRNAs, and circRNAs

**FIGURE 2:**
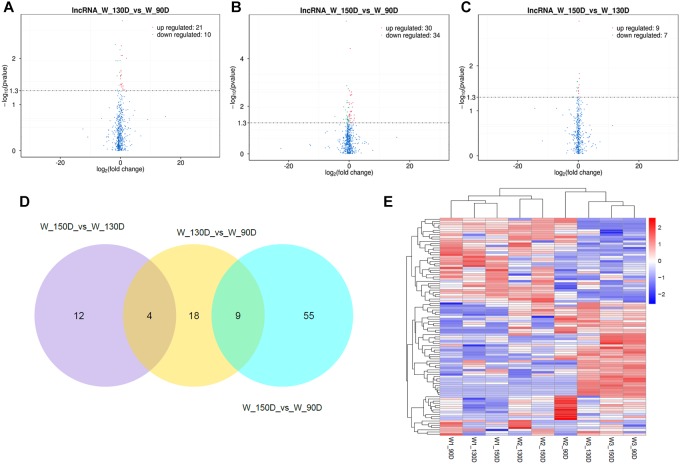
Changes in lncRNA expression during the Angora rabbit hair follicle cycle. **(A–C)** Volcano plots showing up- and down-regulated lncRNAs between days 90, 130, and 150 of the hair follicle cycle. **(D)** Venn diagram showing the number of overlapping differentially expressed lncRNAs between days 90, 130, and 150. **(E)** Heat map of lncRNAs showing hierarchical clustering of DE lncRNAs between days 90, 130, and 150. Up- and down-regulated lncRNAs are shown in red and blue, respectively.

**FIGURE 3:**
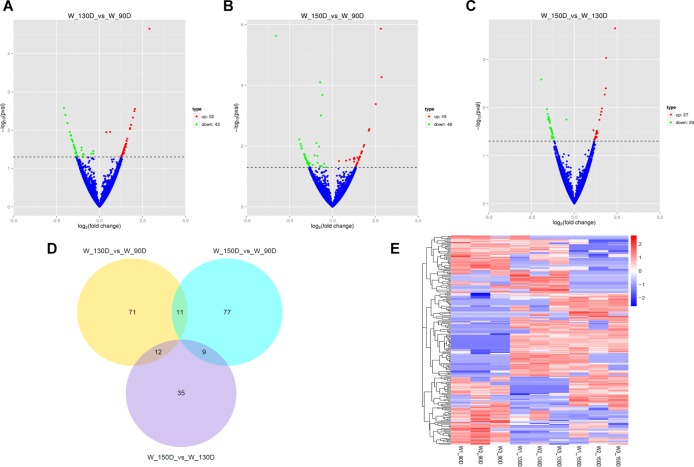
Changes in circRNA expression during the Angora rabbit hair follicle cycle. **(A–C)** Volcano plots showing up- and down-regulated circRNAs between days 90, 130, and 150 of the hair follicle cycle. **(D)** Venn diagram showing the number of overlapping differentially expressed circRNAs between days 90, 130, and 150. **(E)** Heat map of circRNAs showing hierarchical clustering of DE circRNAs between days 90, 130, and 150. Up- and down-regulated circRNAs are shown in red and blue, respectively.

**FIGURE 4:**
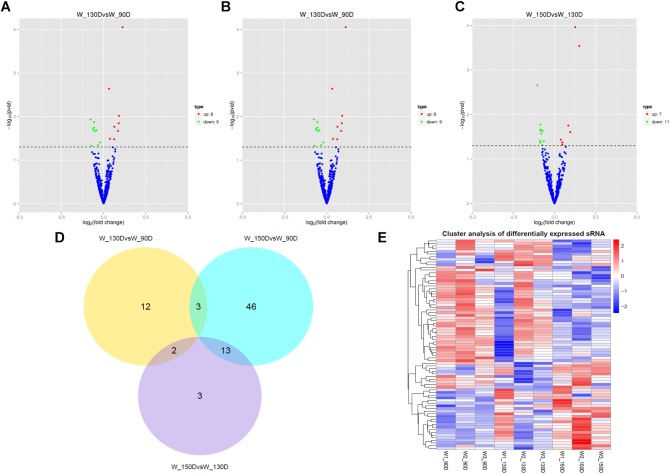
Changes in miRNA expression during the Angora rabbit hair follicle cycle. **(A–C)** Volcano plots showing up- and down-regulated miRNAs between days 90, 130, and 150 of the hair follicle cycle. **(D)** Venn diagram showing the number of overlapping differentially expressed miRNAs between days 90, 130, and 150. **(E)** Heat map of miRNAs showing hierarchical clustering of DE miRNAs between days 90, 130, and 150. Up- and down-regulated miRNAs are shown in red and blue, respectively.

**FIGURE 5:**
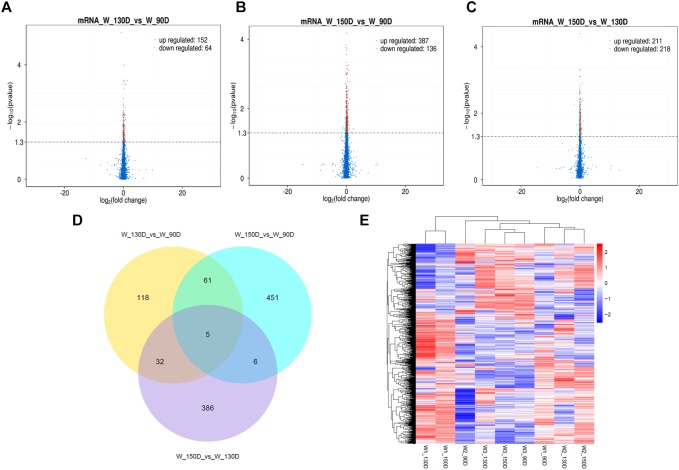
Changes in mRNA expression during the Angora rabbit hair follicle cycle. **(A–C)** Volcano plots showing up- and down-regulated mRNAs between days 90, 130, and 150 of the hair follicle cycle. **(D)** Venn diagram showing the number of overlapping differentially expressed mRNAs between days 90, 130, and 150. **(E)** Heat map of mRNAs showing hierarchical clustering of DE mRNAs between days 90, 130, and 150. Up- and down-regulated mRNAs are shown in red and blue, respectively.

A summary of the lncRNA-seq, miRNA-seq, and circRNA-seq data from the three HF cycle phases is shown in Supplementary Table S3, indicating the relatively high quality of the transcriptome data. The lncRNA-seq, miRNA-seq, and circRNA-seq data were deposited in the Short Read Archive (SRA) of the National Center for Biotechnology Information (NCBI) under the bioproject numbers PRJNA479733, PRJNA495446, and PRJNA495449. DE ncRNAs and mRNAs were analyzed using Cuffdiff software with a criterion of *p* < 0.05. Volcano plots, clustering maps, and Venn diagrams were used to illustrate the distribution of the DE ncRNAs and mRNAs between the three groups (Figure 2–5). Table 1 summarizes the number of DE ncRNAs and mRNAs. Differential expressions of 111 lncRNAs (60 upregulated and 51 downregulated), 247 circRNAs (128 upregulated and 119 downregulated), 97 miRNAs (38 upregulated and 59 downregulated), and 1,168 mRNAs (750 upregulated and 418 downregulated) were found between the three HF cycle stages. Complete information on all DE lncRNAs, circRNAs, miRNAs, and mRNAs is listed in Supplementary Tables S4–S7. Several lncRNAs were found to be associated with the HF cycle, such as LNC_002694, LNC_002919, LNC_003354, LNC_003790, LNC_008354, LNC_008931, and LNC_005484, which could regulate gene expression by recognizing their target mRNAs. Based on analysis of their biological function, the candidate lncRNAs associated with the HF cycle are listed in Supplementary Table S8. Moreover, analysis of the relationships between circRNAs and genes allowed identification of novel_circ_0004876, novel_circ_0005177, novel_circ_0026326, novel_circ_0034968, and novel_circ_0036671, which may play a role during the HF cycle. In addition, several miRNAs, including miR-128-3p, miR-200a-3p, miR-27a-3p, miR-30e-5p, and miR-320-3p; mRNAs, such as *BMP2, CSNK2B, KRT17, LAMB1, FZD4, SMAD2, HTATIP2*, and *SIAH1* were identified to play pivotal roles during the HF cycle and during skin development.

**Table 1:** Summary of the number of differentially expressed ncRNAs and mRNAs.

Groups	Regulation	lncRNA	circRNA	miRNA	mRNA
130 vs. 90 days	Up	21	52	8	152
	Down	10	42	9	64
150 vs. 90 days	Up	30	49	23	387
	Down	34	48	39	136
150 vs. 130 days	Up	9	27	7	211
	Down	7	29	11	218
Total		111	247	97	1168


### Validation of Differentially Expressed lncRNAs, circRNAs, miRNAs, and mRNAs by qPCR

**FIGURE 6:**
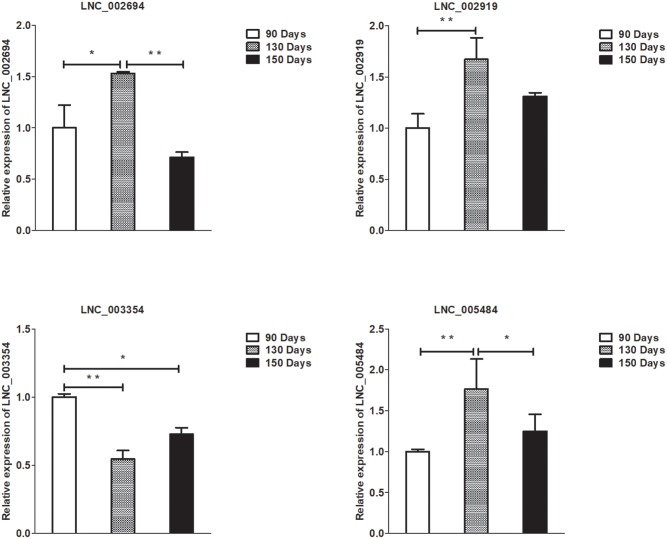
Validation of lncRNA differential expression results at 90, 130, and 150 days. qRT-PCR validation of LNC_002694, LNC_002919, LNC_003354, and LNC_005484 lncRNA expression levels in skin samples between 90, 130, and 150 days. The lncRNA expression levels at 130 and 150 days were normalized to the value at 90 days. Error bars indicate the mean ± SD of triplicate experiments. ^∗^*P* < 0.05; ^∗∗^*P* < 0.01.

**FIGURE 7:**
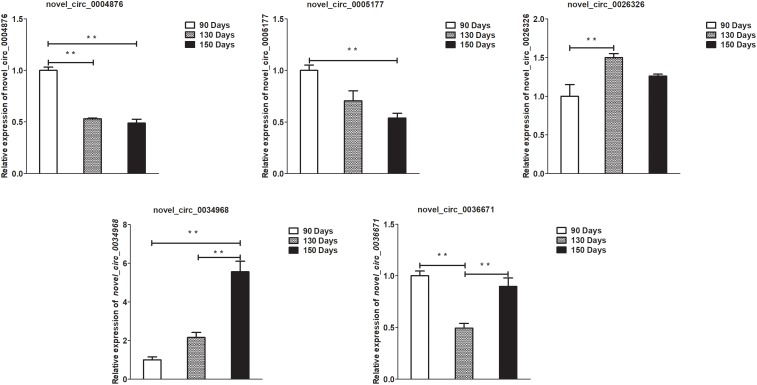
Validation of circRNA differential expression results at 90, 130, and 150 days. qRT-PCR validation of novel_circ_0004876, novel_circ_0005177, novel_circ_0026326, novel_circ_0034968, and novel_circ_0036671 circRNA expression levels in skin samples between 90, 130, and 150 days. The circRNA expression levels at 130 days and 150 days were normalized to the value at 90 days. Error bars indicate the mean ± SD of triplicate experiments. ^∗∗^*P* < 0.01.

**FIGURE 8:**
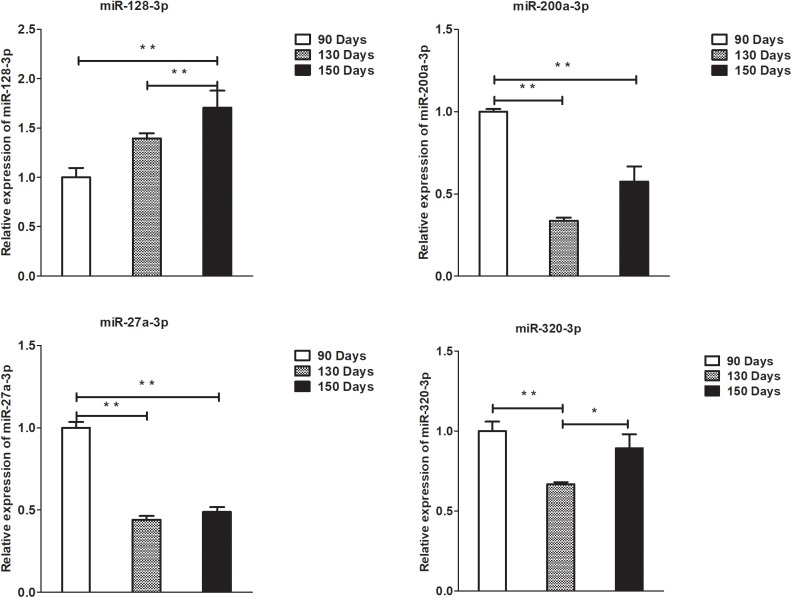
Validation of miRNA differential expression results at 90, 130, and 150 days. qRT-PCR validation of miR-128-3p, miR-200a-3p, 27a-3p, and miR-320-3p miRNA expression levels in skin samples between 90, 130, and 150 days. The miRNA expression levels at 130 days and 150 days were normalized to the value at 90 days. Error bars indicate the mean ± SD of triplicate experiments. ^∗^*P* < 0.05; ^∗∗^*P* < 0.01.

**FIGURE 9:**
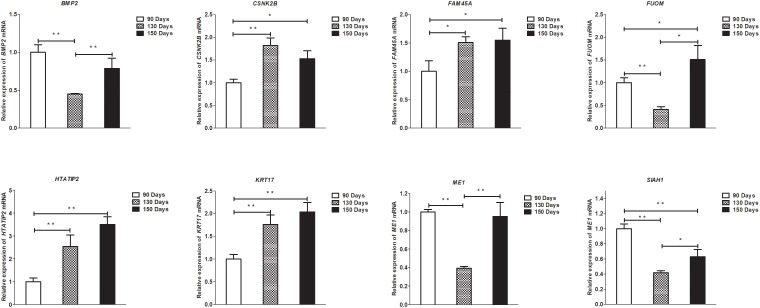
Validation of mRNA differential expression results at 90, 130, and 150 days. qRT-PCR validation of *BMP2, CSNK2B, FAM45A, FUOM, HTATIP2, KRT17, ME1*, and *SIAH1* mRNA expression levels in skin samples between 90, 130, and 150 days. The mRNA expression levels at 130 days and 150 days were normalized to the value at 90 days. Error bars indicate the mean ± SD of triplicate experiments. ^∗^*P* < 0.05; ^∗∗^*P* < 0.01.

To validate the lncRNAs, mRNAs, miRNAs, and circRNAs differential expression results, the relative expression of four DE lncRNAs (LNC_002694, LNC_002919, LNC_003354, and LNC_005484), five DE circRNAs (novel_circ_0004876, novel_circ_0005177, novel_circ_0026326, novel_circ_0034968, and novel_circ_0036671), four DE miRNA (miR-128-3p, miR-200a-3p, miR-27a-3p, and miR-320-3p), and eight DE mRNAs (*BMP2, CSNK2B, FAM45A, FUOM, HTATIP2, KRT17, ME1*, and *SIAH1*) were measured by qRT-PCR (Figure 6–9). The qRT-PCR results were consistent with the transcriptome sequencing data.

### GO and KEGG Pathway Analysis

lncRNAs can regulate neighboring protein-coding genes; therefore, a colocalization threshold of 100 kb upstream or downstream of lncRNAs was set for the GO and KEGG analyses. Several GO terms were found that were significantly enriched in the three experimental groups (Supplementary Table S9), including skin and HF-related GO terms like HF development (GO: 0001942), hair cycle (GO: 0042633), hair cycle process (GO: 0022405), regulation of HF development (GO: 0051797), and skin morphogenesis (GO: 0043589), among others. The top 20 KEGG pathways associated with DE lncRNAs between days 90, 130, and 150 of the HF cycle based on the function of colocalized mRNAs (Supplementary Figure S1) and co-expressed mRNAs (Supplementary Figure S2) included the Wnt signaling pathway, TGF-β signaling pathway, MAPK signaling pathway, and JAK/STAT signaling pathway.

In addition, based on the relationship between circRNAs and genes, GO analysis of genes producing DE circRNAs was performed (Supplementary Table S10). The GO terms identified HF development (GO: 0001942), hair cycle process (GO: 0022405), hair cycle (GO: 0042633), and skin development (GO: 0043588), which were all related to skin and HF development. The top 20 KEGG pathways associated with genes producing DE circRNAs between 90, 130, and 150 days (Supplementary Figure S3) of the HF cycle were likewise related to skin and HF development, such as the Hedgehog signaling pathway, Wnt signaling pathway, and MAPK signaling pathway.

Furthermore, GO enrichment analysis of genes targeted by DE miRNA (Supplementary Table S11) identified GO terms related to HF development, such as HF morphogenesis (GO: 0031069), negative regulation of HF development (GO: 0051799), and regulation of HF development (GO: 0051797), among others. The top 20 KEGG pathways associated with DE miRNAs are shown in Supplementary Figure S4. They include pathways related to HF cycle, such as the Hedgehog signaling pathway, NF-κB signaling pathway, and JAK/STAT signaling pathway.

Finally, GO and KEGG analyses of DE mRNAs are shown in Supplementary Table S12. The GO terms identified include, for example, skin morphogenesis (GO: 0043589) and positive regulation of HF development (GO: 0051798). The top 20 enriched KEGG pathways for DE genes between the different stages of the HF cycle are shown in Supplementary Figure S5. These KEGG pathways include the Wnt signaling pathway, the MAPK signaling pathway, and the TGF-β signaling pathway, which participate in skin development and HF cycle. Differentially expressed genes between days 90, 130, and 150 of the HF cycle, as well as their biological functions, are listed in Supplementary Table S13.

### ceRNA Regulatory Networks

**FIGURE 10:**
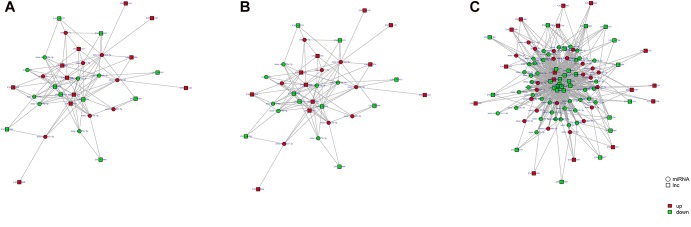
LncRNA–miRNA regulatory networks in Angora rabbit skin. **(A)** Interaction network of lncRNA–miRNA between 130 days and 90 days. **(B)** Interaction network of lncRNA–miRNA between 150 days and 90 days. **(C)** Interaction network of lncRNA–miRNA between 150 days and 130 days.

**FIGURE 11:**
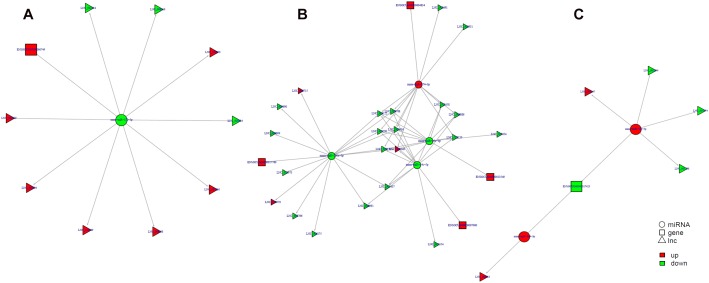
lncRNA–miRNA–mRNA regulatory networks in Angora rabbit skin. **(A)** Interaction network of lncRNA–miRNA–mRNA between 130 days and 90 days. **(B)** Interaction network of lncRNA–miRNA–mRNA between 150 days and 90 days. **(C)** Interaction network of lncRNA–miRNA–mRNA between 150 days and 130 days.

**FIGURE 12:**
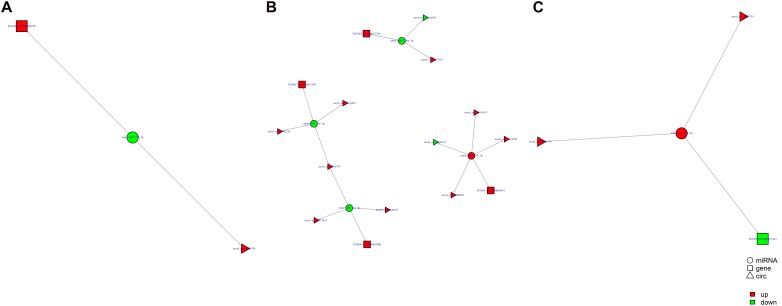
circRNA–miRNA–mRNA regulatory networks in Angora rabbit skin. **(A)** Interaction network of circRNA–miRNA–mRNA between 130 days and 90 days. **(B)** Interaction network of circRNA–miRNA–mRNA between 150 days and 90 days. **(C)** Interaction network of circRNA–miRNA–mRNA between 150 days and 130 days.

Study of the relationship between ncRNAs and mRNAs may increase our understanding of the molecular mechanisms operating during skin development and HF cycle. According to the competing endogenous RNA (ceRNA) regulatory hypothesis, ncRNAs and mRNAs can compete for the same miRNAs, resulting in additional layers of regulation of gene expression. Based on the analysis of DE lncRNAs, circRNAs, miRNAs, and mRNAs, a network of lncRNAs and miRNAs was first constructed (Figure 10). In lncRNA-miRNA-mRNA regulatory networks, miRNA may act as the center, lncRNA as the decoy, and mRNA as the target, which suggests that lncRNAs could act as miRNA sponges to regulate gene expression (Figure 11). In addition, certain circRNAs can competitively bind miRNAs and act as miRNA sponges; therefore, circRNA-miRNA-mRNA triads were constructed with the circRNA as the docoy, miRNA as the center, and mRNA as the target (Figure 12).

**FIGURE 13:**
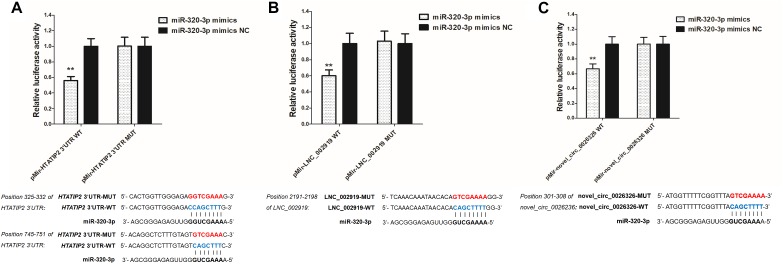
Verification of target binding. **(A)** miR-320-3p putative binding sites in *HTATIP2* 3′-UTR. Blue letters indicate wild type sites and red letters indicate mutated sites in the pMir-report luciferase reporter vector. Luciferase assays were performed in RAB-9 cells co-transfected with pMir-report-HTATIP2-3′-UTR-WT and miRNA-320-3p mimics, or pMir-HTATIP2-3′-UTR-MUT, and miRNA-320-3p mimics. **(B)** Putative binding sites for miR-320-3p in LNC_002919. Blue letters indicate wild type sites and red letters indicate mutated sites in the pMir-report luciferase reporter vector. The luciferase assays were performed in RAB-9 cells co-transfected with pMir-LNC_002919-WT and miRNA-320-3p mimics, or pMir-LNC_002919-MUT, and miRNA-320-3p mimics. **(C)** Putative binding sites for miR-320-3p in novel_circ_0026326. Blue letters indicate wild type sites and red letters indicate mutated sites in the pMir-report luciferase reporter vector. The luciferase assays were performed in RAB-9 cells co-transfected with pMir-novel_circ_0026326-WT and miRNA-320-3p mimics, or pMir-novel_circ_0026326-MUT, and miRNA-320-3p mimics. Error bars indicate the mean ± SD of triplicate experiments. ^∗∗^*P* < 0.01.

LNC_002919 and novel_circ_0026326 were identified as ceRNAs for miR-320-3p, which targets *HTATIP2*. A dual-luciferase reporter system was used to verify the binding relationships between the identified lncRNA and miRNA, circRNA and miRNA, and mRNA and miRNA. Luciferase assay showed that miR-320-3p could decrease luciferase activity by binding to sites on LNC_002919, novel_circ_0026326, and the *HTATIP2* 3′UTR (Figure 13). The interactions between ncRNAs and mRNA suggest the existence of novel regulatory mechanisms during skin development and HF cycle.

## Discussion

The HF cycle is similar in most mammalian species, and many animal models have been used to study the process of hair growth, including mice (Wolbach, 1951; Chase, 1954), rats (Johnson and Ebling, 1964), monkeys (Uno, 1991), cats (Hendriks et al., 1997), and sheep (Hynd et al., 1986). In mice, the hair growth period lasts only 17–19 days, and anterior regions can enter the resting period before the posterior regions regrow (Chase, 1954). By plucking the hairs of rats, the first wave of hair growth was observed between 31 and 22 days, and HF from resting clubs were collected at 55 days of age (Johnson and Ebling, 1964). Although animal HFs show a circannual rhythm, the HF cycles producing sheep wool, horse mane, and human scalp hair have special characteristics, including a biological clock that is independent from day and night, season and temperature over a period of 2–6 years (Stenn and Paus, 2001). The structure, composition, and growth of hair fibers are similar between Angora rabbits and other rabbit breeds. However, the appearance of a mutation in Angora rabbits leads to a prolongation of the anagen phase, so this phase lasts approximately 5 weeks in New Zealand white rabbits but more than 3 months in Angora rabbits (Moore et al., 1987). The HF clock in Angora rabbits has its own characteristic chronobiology, with a long growing period, and independence from seasons and temperature. This study established a synchronization model for hair growth in Angora rabbits. The HFs initiated vigorous growth after shaving the dorsal area, and measuring the length of the hair coat and analyzing the histological characteristics showed that the growth phase lasted about 110 days, the regression period started at about 120 days, and the resting period at about 150 days. The HF synchronization model can contribute to the field of research in the chronobiology of HFs. ncRNAs are epigenetic, translational and genetic regulators that may play a role in numerous biological processes in eukaryotes (Mattick and Makunin, 2006). ncRNAs could play complicated and vital roles during the hair cycle; investigation of the regulatory and functional interactions between lncRNAs, circRNAs, miRNAs, and mRNAs may increase understanding of this biological process.

The present study investigated ncRNAs and mRNAs that were significantly up-regulated or down-regulated during the three stages of the HF cycle. Recent studies have shown that DE lncRNAs modulate biological functions in dermal papilla cells, which regulate postnatal hair cycling and HF cycle (Lin et al., 2015). Likewise, RNA-seq technology has been used for the analysis of lncRNAs and mRNAs during the initiation of sheep secondary HFs (Yue et al., 2016). In addition, miRNAs have been the focus of intense research for several years, and have been associated with HF morphogenesis and development (Mardaryev et al., 2010; Ahmed et al., 2014; Hochfeld et al., 2017). However, only few studies analyzed the involvement of circRNA in skin development and HF cycle. circRNAs can act as miRNA sponges, suppressing miRNA activity and resulting in increased RNA expression (Hansen et al., 2013). This study employed high-throughput sequencing for the analysis of DE ncRNAs in the HF during the different hair cycle stages, based on the synchronization model. A total of 111 lncRNAs, 247 circRNAs, 97 miRNAs, and 1,168 mRNAs were differentially expressed during the hair cycle stages. Moreover, several differentially expressed mRNAs were identified during hair cycling. As a dermal papilla signature gene, *BMP2* is expressed in the hair matrix and can regulate HF cycling (Nakamura et al., 2003; Rendl et al., 2008). In this study, its expression in the catagen was significantly decreased. A previous study reported that KRT17 acts as a key factor to regulate the hair cycling, which affects the transition of anagen-catagen (Tong and Coulombe, 2006). The present results showed that *KRT17* is highly expressed in catagen (via the identified candidates mRNA) between days 130 and 90 as well as between days 150 and 90. Moreover, the co-location relationships between LNC_004603 and *KRT17* were obtained via functional analysis of lncRNA, which indicates that LNC_004603 may act as a potential factor for the regulation of hair cycling. Furthermore, miR-200a-3p is highly expressed in the anagen, which has been proved the be preferentially expressed in the epidermis (Yi et al., 2006). In addition, the expression of miR-128-3p significantly increased from days 90 to 150, with high expression in the telogen. In human HF mesenchymal stem cells, miR-128 could regulate the cell differentiation by targeting SMAD2 (Wang et al., 2016).

Gene ontology analysis includes three domains describing the cellular and molecular roles of genes and gene products (MF, CC, and BP) (Harris, 2004). KEGG is a pathway database for the systematic analysis of gene function, linking genomic and functional information (Ogata et al., 2000). GO and KEGG were used to investigate the potential mechanisms of action of the DE ncRNAs in this study. The obtained results suggest that multiple signaling pathways form a complex regulatory network during skin and HF development. These include the Wnt signaling pathway, the Hedgehog signaling pathway, the TGF-β signaling pathway, the MAPK signaling pathway, the BMP signaling pathway, and the JAK/STAT signaling pathway. These signaling pathways have been previously reported to regulate HF morphogenesis and development (Andl et al., 2002; Mill et al., 2003; Jamora et al., 2005; Kulessa et al., 2014; Akilli Öztürk et al., 2015; Harel et al., 2015). Both *SMAD2* and *SIAH1* were enriched in the Wnt signaling pathway, and *SMAD2* was upregulated at day 150 compared to the differential expression at day 90. In addition, *SIAH1* decreased significantly from days 90 to 130, but increased from days 130 to 150, and was highly expressed when comparing day 150 to day 90. In the cashmere goat, *SIAH1* and *SMAD2* were significantly expressed during the telogen-anagen HF transition. *SIAH1* is highly significantly expressed from telogen to early anagen, and the expression of *SMAD2* increased from telogen to late anagen (Liu et al., 2018). Via functional analysis of lncRNA, the co-expression relationship between LNC_002690 and *SIAH1* was identified, indicating that LNC_002690 might play a central role in hair cycling via regulation of *SIAH1* expression. Hence, these candidates could act as key candidates during HF cycling.

RNA transcripts are regulated by ceRNAs, which compete for the binding of shared miRNAs. miRNA response elements (MREs) are sequences where miRNAs can bind and repress target gene expression. Acting as miRNA sponges, pseudogenes, lncRNAs, circRNAs, and mRNAs can suppress miRNA function through shared MREs (Salmena et al., 2011). Therefore, to try to understand the role of ncRNAs during the HF cycle, lncRNA–miRNA–mRNA and circRNA–miRNA–mRNA regulatory networks were constructed. LNC_002919 and novel_circ_0026326 acted as sponges for miR-320-3p, which targets *HTATIP2*. MiR-320-3p has been reported to either directly or indirectly target genes that regulate the cell cycle and differentiation of the HF (Liu et al., 2013). *HTATIP2* was highly expressed during the catagen and telogen phases, suggesting that *HTATIP2* could inhibit cellular activities during the hair cycle. Decreased or absent HTATIP2 activity modulated through JAK-STAT3 signaling has been shown to play an important role in certain cellular processes. Furthermore, the study shows a link between the JAK-STAT signaling pathway and hair growth (Zhang et al., 2012; Harel et al., 2015). In this analysis of DE lncRNAs, a relationship was found between LNC_002919 and *KRTAP11-1*, suggesting that LNC_002919 could modulate *KRTAP11-1* expression. *KRTAP11-1* influences keratin-bundle assembly and can regulate the physical properties of hair (Fujimoto et al., 2014). Therefore, LNC_002919 could be a potent regulator of the HF cycle. However, the molecular mechanisms underlying the regulation of HTATIP2 by LNC_002919 and novel_circ_0026326, which may act as miR-320-3p sponges, need to be further explored.

## Conclusion

In summary, this study established a rabbit HF synchronization model and investigated the lncRNA, circRNA, miRNA, and mRNA expression profiles by transcriptome analysis of samples collected at different stages of the HF cycle. GO and KEGG pathway enrichment analyses were carried out to identify candidate ncRNAs and mRNAs involved in the regulation of the HF cycle. In addition, ceRNA networks were constructed, which may be active during the HF cycle. These results provide a basis for an improved understanding of the mechanisms underlying the HF cycle.

## Data Availability

The datasets generated for this study can be found in The lncRNA-seq, miRNA-seq, and circRNA-seq data were deposited in the SRA of the NCBI, The lncRNA-seq, miRNA-seq, and circRNA-seq data were deposited in the Short Read Archive (SRA) of the National Center for Biotechnology Information (NCBI) under the bioproject numbers PRJNA479733, PRJNA495446, and PRJNA495449.

## Ethics Statement

The experimental procedures in this study were approved by the Animal Care and Use Committee of Yangzhou University.

## Author Contributions

BZ was responsible for the collection and analysis of results and wrote the manuscript. YC, SH, NY, andMWwere responsible for construction of hair follicle synchronization model. ML, JL, and YX carried out of the experiments. BZ andXWdesigned the study and finalized the manuscript. All authors read and approved the final manuscript.

## Conflict of Interest Statement

The authors declare that the research was conducted in the absence of any commercial or financial relationships that could be construed as a potential conflict of interest.

## Footnotes

1 http://www.geneontology.org

2 http://www.genome.jp/kegg/

## Supplementary Material

FIGURE S1 Top 20 KEGG pathways based on mRNA colocalization with differentially expressed lncRNAs between 90, 130, and 150 days. **(A)** Scatterplot showing KEGG pathway enrichment between 130 and 90 days. **(B)** Scatterplot showing KEGG pathway enrichment between 150 and 90 days. **(C)** Scatterplot showing KEGG pathway enrichment between 150 and 130 days.

FIGURE S2 Top 20 KEGG pathways based on mRNA co-expression with differentially expressed lncRNAs between 90, 130, and 150 days. **(A)** Scatterplot showing KEGG pathway enrichment between 130 and 90 days. **(B)** Scatterplot showing KEGG pathway enrichment between 150 and 90 days. **(C)** Scatterplot showing KEGG pathway enrichment between 150 and 130 days.

FIGURE S3 Top 20 KEGG pathways associated with differentially expressed circRNAs between 90, 130, and 150 days. **(A)** Scatterplot showing KEGG pathway enrichment between 130 and 90 days. **(B)** Scatterplot showing KEGG pathway enrichment between 150 and 90 days. **(C)** Scatterplot showing KEGG pathway enrichment between 150 and 130 days.

FIGURE S4 Top 20 KEGG pathways associated with differentially expressed miRNAs between 90, 130, and 150 days. **(A)** Scatterplot showing KEGG pathway enrichment between 130 and 90 days. **(B)** Scatterplot showing KEGG pathway enrichment between 150 and 90 days. **(C)** Scatterplot showing KEGG pathway enrichment between 150 and 130 days.

FIGURE S5 Top 20 KEGG pathways associated with differentially expressed mRNAs between 90, 130, and 150 days. **(A)** Scatterplot showing KEGG pathway enrichment between 130 and 90 days. **(B)** Scatterplot showing KEGG pathway enrichment between 150 and 90 days. **(C)** Scatterplot showing KEGG pathway enrichment between 150 and 130 days.

TABLE S1 Primers used for the quantitative real-time PCR analysis.

TABLE S2 Primers used for construction of the luciferase reporter vector.

TABLE S3 Summary of RNA sequencing for each sample.

TABLE S4 Analysis of differentially expressed lncRNAs between 90, 130, and 150 days.

TABLE S5 Analysis of differentially expressed circRNAs between 90, 130, and 150 days.

TABLE S6 Analysis of differentially expressed miRNAs between 90, 130, and 150 days.

TABLE S7 Analysis of differentially expressed mRNAs between 90, 130, and 150 days.

TABLE S8 Differentially expressed lncRNAs associated with the hair follicle cycle by target prediction.

TABLE S9 Gene ontology classification of differentially expressed lncRNAs between 90, 130, and 150 days.

TABLE S10 Gene ontology classification of differentially expressed circRNAs between 90, 130, and 150 days.

TABLE S11 Gene ontology classification of differentially expressed miRNA between 90, 130, and 150 days.

TABLE S12 Gene ontology classification of differentially expressed mRNAs between 90, 130, and 150 days.

TABLE S13 Differentially expressed genes between 90, 130, and 150 days of the hair follicle cycle.
