# Supplementary figures and images for: Systematic Analysis of Non-coding RNAs Involved in the Angora Rabbit (Oryctolagus cuniculus) Hair Follicle Cycle by RNA Sequencing
Source: Front Genet. 2019 May 3;10:407. doi: 10.3389/fgene.2019.00407 (PMC6509560; doi:10.3389/fgene.2019.00407)

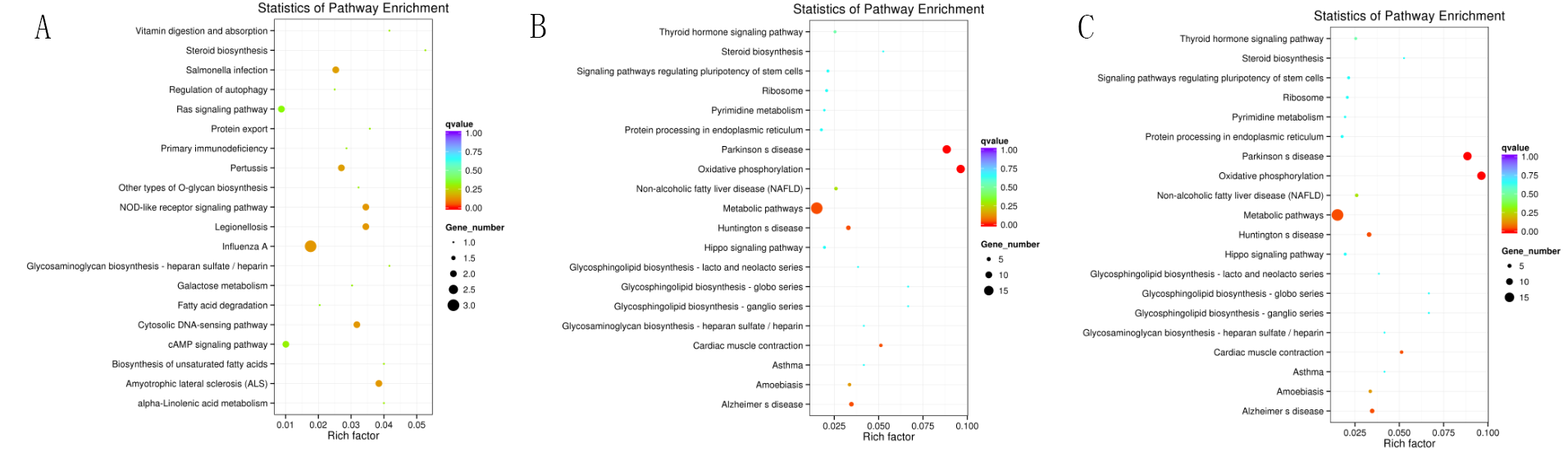

Supplement: FIGURE S1 — Top 20 KEGG pathways based on mRNA colocalization with differentially expressed lncRNAs between 90, 130, and 150 days. (A) Scatterplot showing KEGG pathway enrichment between 130 and 90 days. (B) Scatterplot showing KEGG pathway enrichment between 150 and 90 days. (C) Scatterplot showing KEGG pathway enrichment between 150 and 130 days. [file Image_1.TIF]

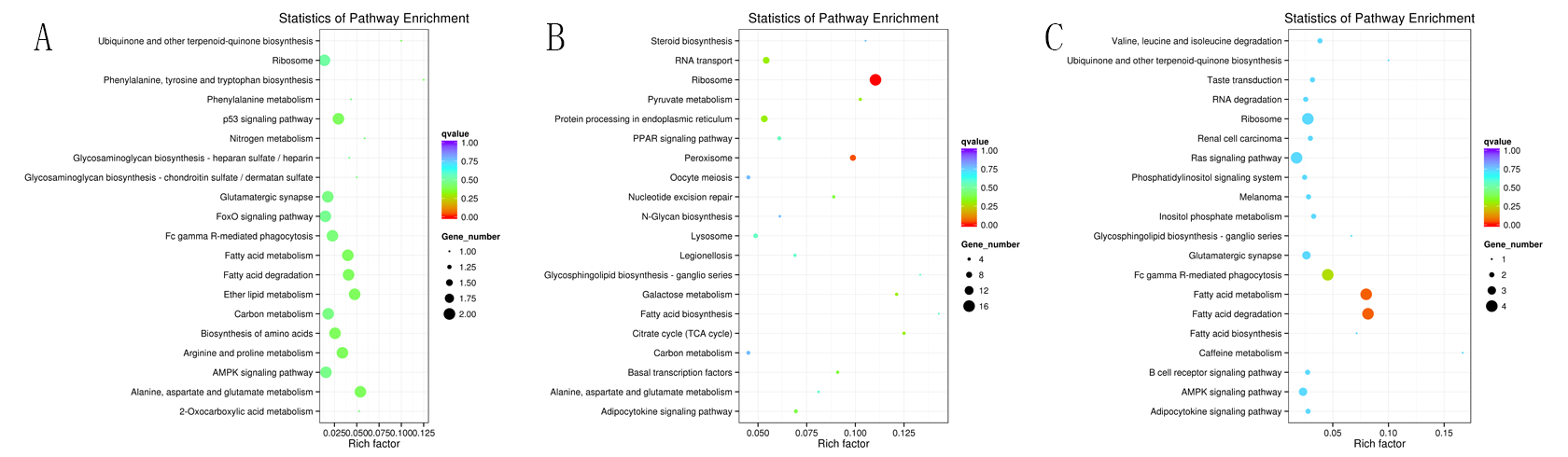

Supplement: FIGURE S2 — Top 20 KEGG pathways based on mRNA co-expression with differentially expressed lncRNAs between 90, 130, and 150 days. (A) Scatterplot showing KEGG pathway enrichment between 130 and 90 days. (B) Scatterplot showing KEGG pathway enrichment between 150 and 90 days. (C) Scatterplot showing KEGG pathway enrichment between 150 and 130 days. [file Image_2.TIF]

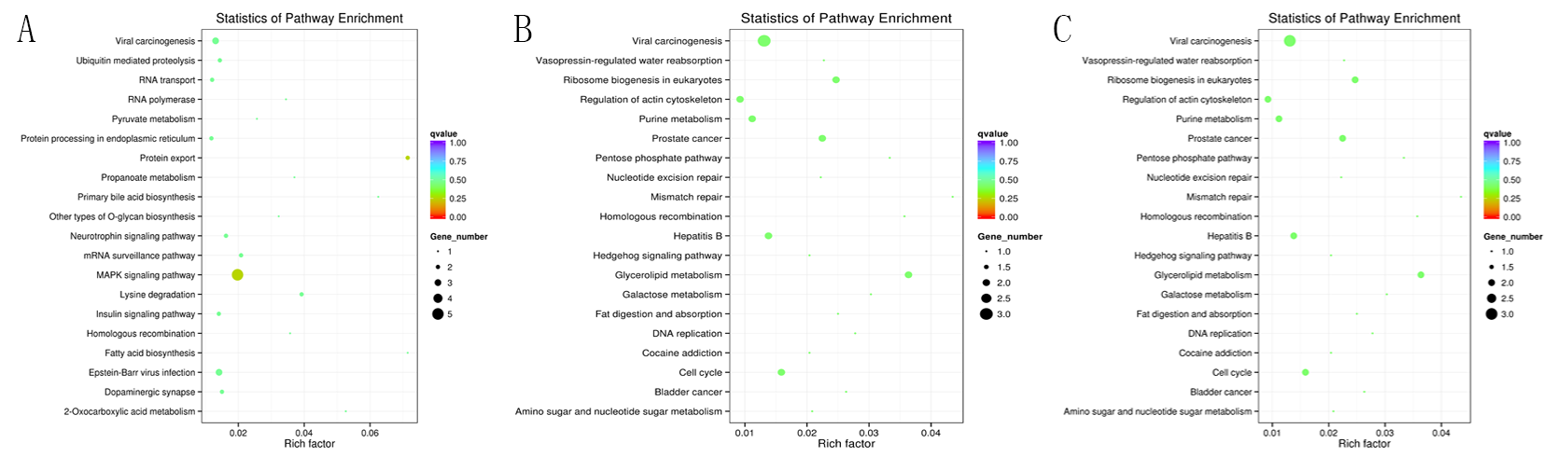

Supplement: FIGURE S3 — Top 20 KEGG pathways associated with differentially expressed circRNAs between 90, 130, and 150 days. (A) Scatterplot showing KEGG pathway enrichment between 130 and 90 days. (B) Scatterplot showing KEGG pathway enrichment between 150 and 90 days. (C) Scatterplot showing KEGG pathway enrichment between 150 and 130 days. [file Image_3.TIF]

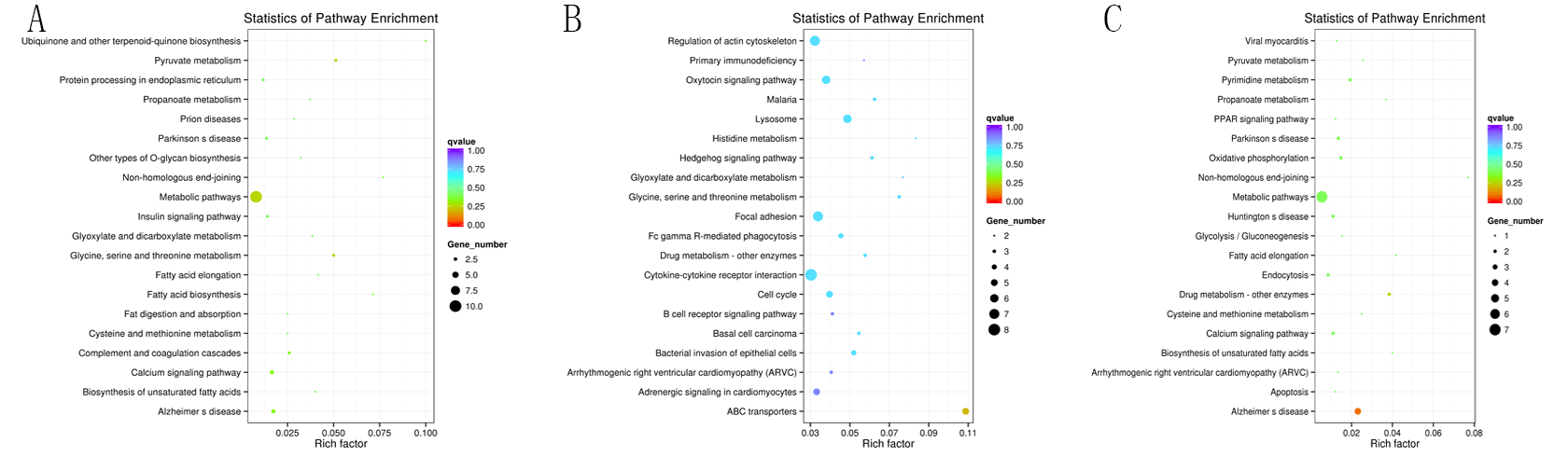

Supplement: FIGURE S4 — Top 20 KEGG pathways associated with differentially expressed miRNAs between 90, 130, and 150 days. (A) Scatterplot showing KEGG pathway enrichment between 130 and 90 days. (B) Scatterplot showing KEGG pathway enrichment between 150 and 90 days. (C) Scatterplot showing KEGG pathway enrichment between 150 and 130 days. [file Image_4.TIF]

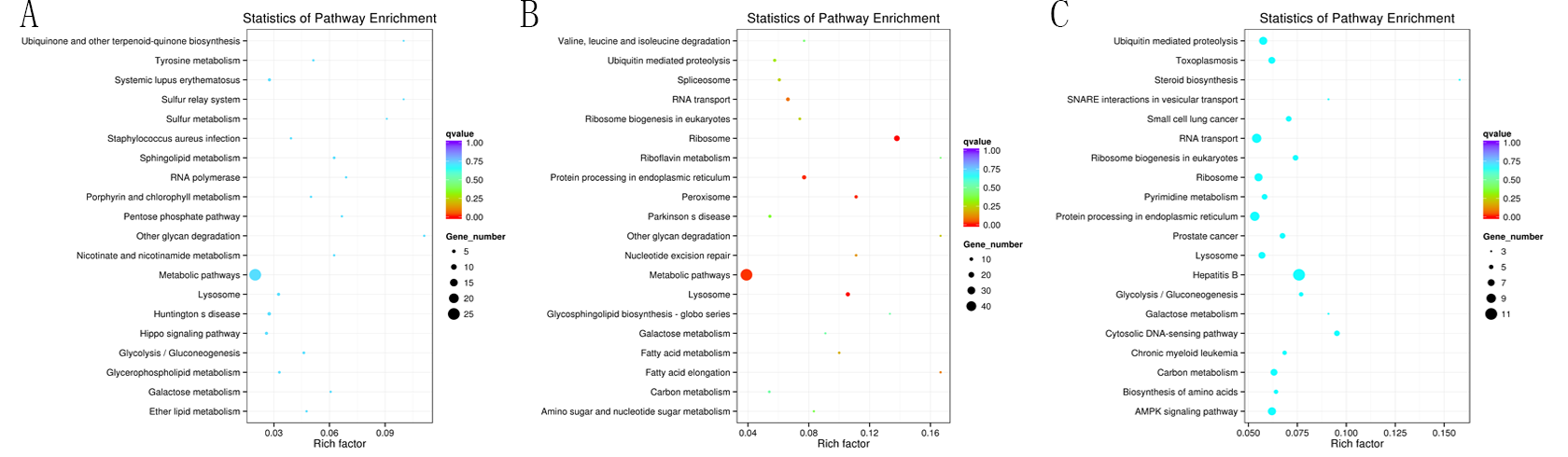

Supplement: FIGURE S5 — Top 20 KEGG pathways associated with differentially expressed mRNAs between 90, 130, and 150 days. (A) Scatterplot showing KEGG pathway enrichment between 130 and 90 days. (B) Scatterplot showing KEGG pathway enrichment between 150 and 90 days. (C) Scatterplot showing KEGG pathway enrichment between 150 and 130 days. [file Image_5.TIF]
